# Development of a broad spectrum glycoconjugate vaccine to prevent wound and disseminated infections with *Klebsiella pneumoniae* and *Pseudomonas aeruginosa*

**DOI:** 10.1371/journal.pone.0203143

**Published:** 2018-09-06

**Authors:** Nicolas Hegerle, Myeongjin Choi, James Sinclair, Mohammed N. Amin, Morgane Ollivault-Shiflett, Brittany Curtis, Rachel S. Laufer, Surekha Shridhar, Jerod Brammer, Franklin R. Toapanta, Ian Alan Holder, Marcela F. Pasetti, Andrew Lees, Sharon M. Tennant, Alan S. Cross, Raphael Simon

**Affiliations:** 1 Center for Vaccine Development and Global Health, University of Maryland School of Medicine, Baltimore, MD, United States of America; 2 Fina Biosolutions, Rockville, MD, United States of America; 3 Department of Microbiology, College of Medicine, University of Cincinnati, Cincinnati, Ohio, United States of America; 4 Department of Surgery, College of Medicine, University of Cincinnati, Cincinnati, Ohio, United States of America; 5 Shriners Burns Institute, Cincinnati, Ohio, United States of America; Instituto Butantan, BRAZIL

## Abstract

*Klebsiella pneumoniae* (KP) and *Pseudomonas aeruginosa* (PA) are important human pathogens that are associated with a range of infection types, including wound and disseminated infections. Treatment has been complicated by rising rates of antimicrobial resistance. Immunoprophylactic strategies are not constrained by antimicrobial resistance mechanisms. Vaccines against these organisms would be important public health tools, yet they are not available. KP surface O polysaccharides (OPS) are protective antigens in animal models of infection. Similarly, PA flagellin (Fla), the major subunit of the flagellar filament, is required for virulence and is a target of protective antibodies in animal models. We report herein the development of a combined KP and PA glycoconjugate vaccine comprised of the four most common KP OPS types associated with human infections (O1, O2, O3, O5), chemically linked to the two Fla types of PA (FlaA, FlaB). Conjugation of KP OPS to PA Fla enhanced anti-polysaccharide immune responses and produced a formulation that generated antibody titers to the four KP OPS types and both PA Fla antigens in rabbits. Passive transfer of vaccine-induced rabbit antisera reduced the bacterial burden and protected mice against fatal intravenous KP infection. Mice passively transferred with conjugate-induced antisera were also protected against PA infection after thermal injury with a FlaB-expressing isolate, but not a FlaA isolate. Taken together, these promising preclinical results provide important proof-of-concept for a broad spectrum human vaccine to prevent KP and PA infections.

## Introduction

Nosocomial infections are a major risk factor upon hospitalization and are associated with a range of infections including those at surgical sites and wounds, pneumonias, intravenous catheter-based infections, urinary tract infections, and septicemia. The recent emergence of antimicrobial resistance (AMR) among nosocomially-associated bacteria, including to antibiotics of last resort such as colistin, engenders significant clinical risk and threatens a return to the pre-antibiotic era. Two Gram-negative bacterial pathogens, *Klebsiella pneumoniae* (KP) and *Pseudomonas aeruginosa* (PA), account for a significant proportion of nosocomial infections and have been identified by the US Centers for Disease Control and Prevention (CDC) and the World Health Organization as being of highest concern due to the accelerating acquisition and spread of antibiotic resistance [1, 2]. Vaccine approaches are not subject to the evasion mechanisms mediating resistance to antibiotics, and thus represent a promising adjunct approach toward ameliorating the burden of AMR bacterial infections [3, 4]. There are, however, no licensed human vaccines for KP and PA. As the clinico-epidemiological patterns for nosocomial infections with these pathogens are similar, a broad spectrum vaccine is warranted and would offer a potentially straightforward way to reduce their combined incidence [1].

Among the different PA antigens that have been assessed as potential vaccinogens, PA flagella proteins are among the best studied, where they have been documented as essential virulence factors and protective antigens in animal models of burn wound and pneumonia infections [5]. Pathogenic PA express a single polar flagellum that is comprised primarily as a multimer of the single protein flagellin (Fla) for which there are only two known serotypes termed FlaA (including sub-forms A1 and A2) and FlaB [6]. Antibodies directed against Fla proteins have arrested motility, and protected mice against fatal PA infection when induced by active immunization or passively transferred [7, 8]. As immunity has been found to be serotype-specific to either FlaA or FlaB, a broadly protective PA vaccine would require coverage for both antigens [7].

While comparatively less effort has been directed towards development of a KP vaccine due to historical susceptibility to antibiotics, preclinical studies have documented the capacity of antibodies directed against KP surface polysaccharides to protect against lethal KP challenge in animal models [9]. KP can express both a capsule polysaccharide (K antigen) and a lipopolysaccharide-associated O polysaccharide (OPS) on their surface. There are, however, greater than 80 known KP capsular serotypes for which no single K type predominates and notable geographical disparities exist for the most common circulating types [10, 11]. It has thus been estimated that a broadly protective vaccine would require coverage for at least 24 different K types representing a notable barrier to the development of a capsule-based KP vaccine [10, 11]. By comparison, only 9 KP OPS structures have been described, for which a subset (O1, O2, O3, O5) are associated with the vast majority (>80%) of clinical isolates worldwide [10, 12–14]. Despite the presence of a capsule, KP OPS has been found to be accessible to antibodies that have been shown to mediate opsonophagocytic uptake with killing by oxidative burst, and imparted protection in animal models of pneumonic and disseminated infection [15–17]. As isolated antigens, bacterial polysaccharides are generally poorly immunogenic and fail to induce immunological memory, a limitation that is overcome by linkage or association with a protein carrier [18]. We report, herein, the preclinical development of a broad-spectrum four component conjugate vaccine for KP and PA based on chemical conjugation of the four prevalent KP OPS types with the two PA flagellin types.

## Materials and methods

### Bacterial strains, medium, growth and typing

The strains used in this study are described in Table 1. For general maintenance, *K*. *pneumoniae* and *P*. *aeruginosa* were grown at 37°C overnight in Hy-Soy (HS) bacteriological media (5 g/L sodium chloride, 10 g/L soytone [Teknova, CA], 5 g/L Hy-yest [Sigma Aldrich, MO]) at 37°C. 1.5% bacteriological agar (AmericanBio, MA) was added to HS for solid media preparations. Fully chemically defined medium (CDM) used for growth of KP in fermentation cultures and to assess guanine auxotrophy has been described previously [19]. 0.004%-0.025% guanine (Sigma Aldrich, MO) was added for the growth of CVD 3001 to supplement the *guaBA* mutation. O types were determined by PCR with extracted genomic DNA as described [14]. K types were determined by sequencing of the *wzi* or *wzc* genes, or multiplex PCR [20–22].

**Table 1:** List of bacterial strains used in this study.

Species	Strain	Source/characteristics (use)	Reference
*K*. *pneumoniae*	B5055	O1:K2 (challenge strain)	[23]
*K*. *pneumoniae*	7380	O2:K- (reagent strain)	[24]
*K*. *pneumoniae*	390	O3:K11 (reagent strain)	[24]
*K*. *pneumoniae*	4425/51	O5:K57 (parent strain for CVD 3002)	[24]
*K*. *pneumoniae*	700603	O3:K-undetermined (challenge strain)	ATCC
*K*. *pneumoniae*	B5055 Δ*guaBA*	Precursor to CVD 3001, used for guanine auxotrophy confirmation.	This study
*K*. *pneumoniae*	CVD 3001	B5055 Δ*guaBA* Δ*wzabc* (reagent strain)	This study
*K*. *pneumoniae*	CVD 3002	4425/51 Δ*wzabc* (reagent strain)	This study
*K*. *pneumoniae*	15–03671	Clinical isolate; O1:K1 (flow-cytometry)	J. Johnson, University of Maryland Medical Center (UMMC)
*K*. *pneumoniae*	081091	Clinical isolate; O1:K3 (flow-cytometry)	J. Johnson, UMMC
*K*. *pneumoniae*	1200428	Clinical isolate; O1:K12 (flow-cytometry)	J. Johnson, UMMC
*K*. *pneumoniae*	170381	Clinical isolate; O1:K22 (flow-cytometry)	J. Johnson, UMMC
*K*. *pneumoniae*	1403436	Clinical isolate; O1:K23 (flow-cytometry)	J. Johnson, UMMC
*K*. *pneumoniae*	12–04802	Clinical isolate; O1:K24 (flow-cytometry)	J. Johnson, UMMC
*K*. *pneumoniae*	60331	Clinical isolate; O1:K28 (flow-cytometry)	J. Johnson, UMMC
*K*. *pneumoniae*	151661	Clinical isolate; O1:K38 (flow-cytometry)	J. Johnson, UMMC
*K*. *pneumoniae*	120581	Clinical isolate; O1:K62 (flow-cytometry)	J. Johnson, UMMC
*E*. *coli*	BL21 p*Trc-flaA*	rFlaA reagent strain	This study
*E*. *coli*	BL21 p*Trc-flaB*	rFlaB reagent strain	This study
*P*. *aeruginosa*	PAO1	FlaB (motility inhibition)	[25]
*P*. *aeruginosa*	PAK	FlaA1 (challenge strain, motility inhibition)	[26]
*P*. *aeruginosa*	M2	FlaB (challenge strain)	[8]

### Genetic engineering

Genomic DNA was purified using the DNeasy Blood and Tissue kit (Qiagen, MD) or the GenElute Bacterial Genomic DNA kit (Sigma-Aldrich, MO). Plasmids were purified using the Wizard® Plus SV Minipreps DNA purification kit (Promega, WI). The primers used in this study are listed in S1 Table. PCR was performed using Vent^®^ DNA polymerase (New England Biolabs, MA) according to the manufacturer’s protocol. PCR products were purified using the QIAQUICK PCR Purification Kit (Qiagen, MD) according to the manufacturer’s protocol.

#### (i) Reagent strains for KP OPS production

Deletion of *guaBA* and *wzabc* in *K*. *pneumoniae* was achieved by lambda red-mediated mutagenesis [27] using a linear DNA cassette constructed by the overlapping PCR method based on that described by Chalker et al. [28] and adapted by Tennant et al. [29]. The primers used in this study are described in S1 Table. Genetic deletions were confirmed by sequencing the scar region remaining after excision of the antibiotic resistance gene. Guanine auxotrophy was confirmed as described previously by comparing the growth of the parental strain and the mutant on a fully chemically defined medium with or without addition of 0.01% guanine [29]. Confirmation of loss of capsule was carried out by negative stain using India Ink (Hardy Diagnostics, CA). Briefly, a single drop of India ink was placed on a clean microscope glass slide. A single colony from an overnight isolation of the wild-type or mutant strain was mixed in the drop, which was then spread out and covered with a glass cover slip. The preparation was observed under a light microscope at a final 1000X magnification with a 100X oil immersion objective.

#### (ii) Reagent strains for PA flagellin production

Synthetic genes encoding A-type (GenBank accession no. CP020659.1) flagellin from PAK and B-type (GenBank accession no. AE004091.2) flagellin from PAO1 were codon optimized by GenScript (NJ) for expression in *E*. *coli* (S2 Table). Both synthetic genes were cloned into pTrc-His-TOPO (Thermo Fisher, MA) after PCR amplification using primers fla_F and fla_R (S1 Table). The resulting plasmids, pTrcHis-*flaA*_*opt*_ and pTrcHis-*flaB*_*opt*_, were then transformed into *E*. *coli* BL21 cells (New England Biolabs, MA) according to the manufacturers protocol, for production of A-type and B-type flagellin, respectively, as recombinant proteins incorporating a 6xHis tag.

### KP OPS production, purification and characterization

KP O1, O2, O3 and O5 reagent strains were grown in 4 L fermentation cultures in a BioStat A+ fermenter (0.6–5 L working volume, 6.6 L total volume) (Sartorius, Germany) essentially as described [19]. Briefly, 5–10 colonies were selected from an HS agar plate grown overnight at 37°C and used to inoculate a 250 mL shake flask containing 50 mL of CDM (supplemented with 0.025% guanine for CVD 3001). This culture was grown for 11–13 h at 37°C with agitation at 250 rpm and then used to inoculate a separate 500 mL culture in a 2 L shake flask containing the same media that was then grown under the same conditions for 11–13 h. Four liters of CDM (with 0.025% guanine for CVD 3001) was then inoculated to an OD_600_ nm of 0.15 with the 500 mL shake flask and maintained in fermentation culture at 400 rpm, 4 LPM ambient air, and continuous adjustment to pH 7 with 28% ammonium hydroxide. Eighteen to 24 hours post inoculation, the whole fermentation culture was then transferred to glass bottles and KP OPS was released by nitrous acid deamination essentially as described previously [13] by bringing the culture to 5–10% acetic acid / 0.5% sodium nitrite pH 3.7 with incubation at 4°C under agitation for 16–24 hours. Insoluble material and bacterial cells were then removed by 10,000 *x g* centrifugation at 4°C with a GS3 Rotor in a Sorvall RC5B. The post-extraction supernatant (PES) was then brought to 1M NaCl and filtered by microfiltration through a 0.2 μm hollow-fiber filter (GE, NJ) at 4.5 psi transmembrane pressure (TMP) passing the full volume followed by flushing with an equivalent volume of 1 M NaCl. Filtered PES / 1 M NaCl was then concentrated ~10-fold on a 0.1 m^2^ 10 kDa Tangential flow filtration (TFF) Hydrosart flat sheet membrane (Sartorius, Germany) at 14 psi TMP and diafiltered 35-fold with 1 M NaCl and then 10-fold with 20 mM Tris / 50 mM NaCl pH 7. The TFF retentate was then passed through a Sartobind Q capsule (75 mL) at 45 mL/min in negative mode with collection of the flow through and a subsequent flush with 5 bed volumes (BV) of 20 mM Tris / 50 mM NaCl pH 7. The pooled flow-through and wash were then brought to 1.9 M Ammonium sulfate and incubated 16–24 hours at 4°C, followed by centrifugation at 10,000 *x g* at 4°C with a GS3 Rotor in a Sorvall RC5. The cleared supernatant was then filtered through 0.45 μm followed by TFF through a 50 cm^2^ 300 kDa polyethersulfone (PES) membrane (Biomax, Millipore, MA) at <2 psi TMP followed by flushing with 60 mL of DI water. Filtered OPS was then concentrated to 5–10 mg/mL by TFF on 2 x 200 cm^2^ 5 kDa Hydrosart membranes in a Slice 200 holder at 14 psi TMP followed by 10-fold diafiltration with DI water. The retentate fraction was then filtered through 0.2 μm, frozen and lyophilized for dry weight recovery. Purified polysaccharides were assessed for removal of protein and endotoxin respectively by Bradford assay (Biorad, CA) and chromogenic limulus amebocyte lysate (LAL) assay with the Endosafe PTS system (Charles River, MA). Residual nucleic acid was assessed by absorbance at 260 nm. Identity for each polysaccharide was confirmed by high performance anion exchange chromatography with pulsed amperometric detection (HPAEC-PAD) with an ICS-4000 system (Thermo-Dionex, MA) and CarboPac PA10 column (Thermo-Dionex, MA) run at 0.01 mL/min using commercially available monosaccharide standards (Sigma, MA). For this, polysaccharide samples and monosaccharide standards were prepared by boiling for 4 hours in 2 M trifluoroacetic acid that were then frozen, lyophilized and reconstituted in milli-Q water for injection. Molecular size was assessed by high performance liquid chromatography with size exclusion chromatography (HPLC-SEC) with a Biosep SEC4000 column (Phenomenex, CA) run at 1 mL/min on an Alliance 2795 HPLC (Waters, MA) in PBS / 0.02% sodium azide pH 7.4 with detection by refractive index (RI) on a 2414 RI detector (Waters, MA).

#### Production and purification of PA rFlaA and rFlaB

PA A-type and B-type flagellin proteins were produced in 8 L fermentation cultures of *E*. *coli* BL21 with p*Trc-flaA* or p*Trc-flaB* in a BioFlo415 bioreactor (4–10 L working volume, 14 L total volume) (Eppendorf, Germany). Briefly, 5–10 single colonies were selected from overnight growth at 37°C on HS plates supplemented with 50 μg/mL carbenicillin for plasmid maintenance, and used to inoculate a 50 mL culture in a 250 mL shake flask containing Terrific Broth (TB; Invitrogen, MA) supplemented with 50 μg/mL carbenicillin and 1% glucose that was grown for 8 hours at 30°C with 250 rpm agitation. This culture was then used to inoculate a separate 500 mL culture in a 2 L shake flask containing the same media that was then grown under the same conditions for 12h. This culture was then used to inoculate the 8 L fermenter culture to an OD_600nm_ of ~0.15, that was grown in TB with 50 μg/mL carbenicillin without glucose at 30°C and maintained at 250 rpm, 4 LPM ambient air, and continuous adjustment to pH 7 with 28% ammonium hydroxide until the OD_600nm_ reached ~3. Induction of flagellin production was then carried out for 90 minutes at 37°C with addition of 1 mM Isopropyl β-D-1-thiogalactopyranoside (IPTG, Sigma Aldrich), after which cells were centrifuged at 10,000 *x g* for 30 min at room temperature and the pellet stored at -80°C until protein extraction. The recombinant flagellin was purified from the soluble fraction after extraction with B-PER reagent (ThermoFisher, MA) according to the manufacturer’s protocol. After centrifugation at 10,000 *x g* for 40 mins at 4°C the supernatant was filtered through a Polycap 150 TC 0.45 μm depth filter (Whatman, UK). The filtered sample was brought to 20 mM Tris, 0.5 M NaCl, 20 mM Imidazole, pH 7.5 (binding buffer) and incubated overnight at 4°C under agitation with Ni-Sepharose™ 6 Fast Flow resin (GE Biosciences, NJ). The resin was poured into a gravity flow column, washed with five BV of binding buffer and the 6xHis recombinant protein eluted with 20 mM Tris / 0.5M NaCl / 250 mM imidazole pH 7.4 with gravity flow. The eluate was then further purified by sequential TFF, cation and anion exchange membrane chromatography as described previously [30]. Purified flagellin proteins were confirmed for purity and integrity by SDS-PAGE with Coomassie staining, removal of residual endotoxin by limulus amebocyte lysate assay with the Endosafe PTS system (Charles River, MA), and were stored at -80°C until use. Protein concentration was assessed by absorbance at 280 nm in a UV spectrophotometer (Beckman, MA) with the respective calculated extinction coefficient for FlaA or FlaB.

#### Synthesis of glycoconjugates by chemical conjugation

KP OPS was chemically linked at the reducing end 2’5’-anhydromannose aldehyde [13] to lysine residues on PA rFlaA or rFlaB proteins with thioether-aminooxy chemistry as described previously [19, 31]. Briefly, KP OPS were brought to 20 mg/mL in 100 mM sodium acetate pH 5 with 2.5 mg/mL of O-(3-mercaptopropyl)-hydroxylamine (Fina Biosolutions, MD). The reaction was incubated for 12–18 hours at room temperature with stirring, at which point 400 mg/mL of sodium cyanoborohydride (Sigma, MA) in water was added at a 2:1 wt:wt ratio with OPS. The reaction was incubated for 3 hours and then brought to 100 mM DTT and purified by desalting through Sephadex G25 in PBS / 5 mM EDTA, pH 6.8. Separately, PA flagellin proteins were brought to 2–4 mg/mL in 10 mM sodium phosphate pH 7.4 with 1 mM N-γ-maleimidobutyryl-oxysuccinimide ester (GMBS, Molecular BioSciences, CO) and incubated for 1 h at room temperature at which point they were then brought to 2–3 mg/mL by TFF with 10 kDa PES hollow-fiber filters (Biomax, Millipore, MA) and diafiltered against 10 diavolumes of PBS / 5 mM EDTA, pH 6.8. Aminooxy-thiol labeled KP OPS was then immediately added at a 1:1 wt:wt ratio to maleimide derivatized PA flagellin and incubated for 24–48 hours at 4°C with tumbling rotation. Conjugation reactions were monitored by SDS-PAGE with Coomassie staining and HPLC-SEC with RI detection. The reaction was then concentrated ~3-fold with a 30 kDa spin filter (Amicon, Thermo, MA), and unreacted protein and polysaccharides removed by separation on a Superdex 200 16/600 column run at 1 mL/min in 20 mM Tris pH 7.4 on a NGC chromatography system (Biorad, CA) with monitoring for absorbance at 280 nm and 260 nm. Fractions containing conjugate were identified by SDS-PAGE with Coomassie staining, pooled and filtered through 0.2 μm, and stored at 4°C until use. Polysaccharide and protein content in purified conjugates was assessed by BCA assay and resorcinol assay [32] with unconjugated protein and polysaccharide standards respectively.

#### Toll-like receptor 5 (TLR5) activity assay

TLR5 assays were conducted as described [33]. Briefly, human epithelial kidney cells stably expressing the firefly luciferase under control of an NF-κB-dependent promoter (HEK293-Luc) were maintained in complete RPMI (Invitrogen, Carlsbad, CA). Cells were maintained at 37°C in a 5% CO_2_ atmosphere; monolayers of 1 x 10^5^ cells per well in a 96-well plate were treated for 4 h with media alone or different dilutions of rFlaA or rFlaB proteins alone or conjugated to KP O1 OPS. Cell extracts were prepared and luciferase activity was measured using the luciferase assay system (Promega, WI) according to the manufacturer's protocol using a LMAX II^384^ (Molecular Devices). The EC_50_ of the carrier protein and conjugates were determined by plotting the logarithm of the protein agonist concentration against the measured luminescence expressed in relative luminescence units (RLU).

### Ethics statement

All animal studies were performed in facilities that are accredited by the Association for Assessment and Accreditation of Laboratory Animal Care and were in compliance with guidelines for animal care established by the US Department of Agriculture Animal Welfare Act, US Public Health Service policies, and US federal law. All animal experiments were approved by the University of Maryland School of Medicine Institutional Animal Care and Use Committee (mice, protocol #0517010) or the Cocalico Biologicals Animal Care and Use Committee (rabbits). Experiments were performed with appropriate administration of anesthesia and analgesics, and all efforts were made to minimize suffering. Animals were housed in accredited animal facilities, and provided with suitable environmental enrichment, and fresh water and feed *ad libitum*. They were monitored multiple times per week for overall health, and daily for mice that had undergone bacterial challenge. Mouse anesthesia was by parenteral ketamine/xylazine or inhaled isoflurane, and euthanasia was accomplished by carbon dioxide asphyxiation with confirmation by cervical dislocation. Bacterial clinical isolates data had been de-identified and were analyzed anonymously.

### Animal studies

#### Mice and rabbit immunization

Six-to 7-week-old female Crl:CD-1 mice (Charles River Laboratories, MA) were injected intramuscularly (IM) in the right gastrocnemius at 0, 14 and 28 days with either sterile PBS pH 7.4, the O1:rFlaA conjugate vaccine at 5 μg O1 OPS/dose or the O1:rFlaB conjugate vaccine at 2.5 μg O1 OPS/dose. Serum was obtained by centrifugation of blood collected by retro-orbital bleeding in Z-gel Micro tubes (Sarstedt, Germany) before the first immunization and two weeks after the third vaccination. Serum was stored at -20°C until use. New Zealand White rabbits were immunized under standard protocols at Cocalico Biologicals (PA) by IM injection at 0, 14, 28 and 42 days with quadrivalent conjugate (formulated at 5 μg of each polysaccharide per dose), admixed unconjugated purified OPS and recombinant flagellin molecules (5 μg of each respective OPS and flagellin component), 5 μg rFlaA, or 5 μg rFlaB proteins, with the first dose formulated in complete Freunds adjuvant and the remaining doses formulated in incomplete Freunds adjuvant. Sera were obtained prior to the first dose, 14 days after the third dose and 28 days after the 4^th^ immunization, and stored at -20°C until use.

#### Intraperitoneal (IP) LD_50_ studies

Six-to 7-week-old female Crl:CD-1 mice were infected IP with different doses of KP B5055 or CVD 3001 or by burn wound infection as described below with PA M2, or PAK. Mortality after infection was assessed for 1 week recording weight loss and overall health. Mice that had lost ≥ 20% body weight and appeared moribund were euthanized and recorded as having succumbed to infection. LD_50_ values were determined by the method of Reed and Muench [34].

#### Passive transfer protection studies for invasive KP and PA burn wound infections

Six-to 7-week-old female Crl:CD-1 mice were given three doses of pre-immune or conjugate-immunized pooled rabbit sera (taken after the 4^th^ dose) via IP administration 24 and 2 hours before challenge and 24 hours after challenge. For KP challenge, mice were challenged as described previously [15] by administration of ~9 x 10^4^ KP colony forming units (CFU) intravenously (IV) in 100 μL of sterile PBS through the tail vein. Bacterial clearance was assessed 4 hours after infection by assessing viable CFU in the blood, liver and spleen. Mortality after IV KP infection was assessed for 1 week as described above. For PA burn wound infection, mice were infected as described with minor modifications [35]. Briefly, mice had their backs shaved 24 hours prior to infection. On the day of infection, they were sedated with ketamine and xylazine and then the burn was induced by pressing a polymer card cutout template representing roughly 30% of total body surface area on the shaved back followed by the application of 0.5 mL of 90% ethanol spread uniformly into the cutout, ignited with a flame and allowed to burn for 10 seconds. Mice were infected by subcutaneous injection at the burn site with 100 μL of either ~10–20 CFU of PA M2 (LD_50_ <10 CFU) or ~3–4 x 10^6^ CFU of PA PAK (LD_50_ ~3.5 x 10^5^ CFU) in PBS, and replenished with 500 μL sterile saline given IP. A separate group of bystander mice were burned but not infected to confirm survival from the burn wound procedure alone. All mice were monitored daily for up to 14 days after challenge as described above. Mice that reached a moribund state were euthanized and recorded as dead. Vaccine efficacy (VE) was calculated as VE = ([mortality in control group]–[mortality in group receiving serum]) / (mortality in control group) *100.

### Enzyme-linked immunosorbent assay (ELISA)

Serum IgG was measured by ELISA. For anti-rFla IgG titration, clear round-bottom immune nonsterile 96-well plate (Immulon 2 HB Thermo Scientific, MA) were coated with 100 μL/well rFlaA at 1.0 μg/mL or 100 μL/well rFlaB at 1.0 μg/mL in PBS, pH 7.4. For anti-OPS IgG titration, biotinylated KP OPS was prepared as capture antigen by incubation of 10 mg/mL KP OPS with amino-PEG_3_-biotin (EZ Link, Thermo, MA) for 18–24 hours followed by addition of 200 mg/mL sodium cyanoborohydride for an additional 18–24 hours, at which point the OPS-biotin was purified by dialysis with 3 kDa cassettes (Thermo, MA) with DI water. Immulon 2 HB flat-bottom 96-well plates were coated with 3.0 μg/mL Streptavidin (Millipore Sigma, MO) overnight at 37°C. Biotinylated OPS antigens were added 100 μL/well at 2.0 μg/mL for 3h at 37°C followed by 6 washes with PBS + 0.05% Tween 20 (PBS-T). Plates were then blocked with PBS, pH 7.4, with 10% Non-Fat Dry Milk overnight at 4°C. Mouse or rabbit serum samples diluted in PBS-T +10% Non-Fat Dry Milk, pH 7.4 were added in duplicate wells and incubated for 1 h at 37°C, followed by 6 washes with PBS-T. Bound mouse or rabbit IgG was detected with HRP-labeled Goat anti-Mouse IgG (Seracare, MD) or HRP-labeled Goat anti-Rabbit IgG (Life Technologies, CA) respectively, diluted 1:1,000 and 1:2,000 in 10% Non-Fat Dry Milk in PBS-T, pH 7.4, for 1h at 37°C. After washing, substrate (3,3’,5,5’-tetramethylbenzidine, Seracare, MD) was added, and the plates were incubated on a rocker at ambient temperature for 15 min in darkness. The reaction was stopped with the addition of 1 M H_3_PO_4_, and the absorbance at 450 nm was recorded using a Multiskan FC^TM^ Microplate Reader (Thermo, MA). Test and control sera were run in duplicate. Titers were calculated by interpolation of absorbance values of test samples into the linear regression curve of a calibrated control (reference serum). The endpoint titers reported as ELISA units (EU) represent the inverse of the serum dilution that produces an absorbance value of 0.2 above the blank.

### Opsonophagocytic uptake assay (OPA)

J774 mouse macrophage cells [36] were obtained from the ATCC and uptake of KP into these cells was determined essentially as described previously [32]. Briefly, KP bacteria (B5055 [O1] or 700603 [O3]) were grown overnight in Hy-Soy media at 37°C / 250 rpm, harvested by 15k x G centrifugation and washed with PBS. Approximately 10^5^ bacteria were then incubated with heat-inactivated (56°C for 30 mins) pre-immune or conjugate-induced rabbit sera (obtained after the 4^th^ dose) for 30 mins at room temperature with gentle agitation. The opsonized organisms were then added to J774 cells seeded in DMEM with 10% fetal bovine serum (FBS, Sigma, MA) at 10^5^ cells per well in 24-well tissue culture plates and incubated at 37°C, 5% CO_2_. One hour later, the media was replaced with DMEM + 10% FBS supplemented with 100 μg/mL gentamicin, and the plates were then incubated for an additional hour at 37°C, 5% CO_2_, at which point the J774 monolayers were washed three times with sterile PBS, lysed with 0.5% Triton X-100 (Sigma, MA), and viable CFU determined by plating on solid agar growth medium. As negative controls, bacteria were incubated with media alone.

### Motility inhibition assay

For motility inhibition assays, *P*. *aeruginosa* strains were grown overnight at 37°C / 250 rpm in Hy-Soy medium to stationary phase. The cells were pelleted at 10k x G, washed in PBS, resuspended in PBS and adjusted to an OD_600_ of 0.1. Normalized suspensions were diluted 1:1,000 in PBS and incubated alone or with 1:30 diluted sera for 30 minutes at 37°C. A sterile wire dipped in the bacterial preparation was then used to spike inoculate the bacteria ~2mm deep in a 0.3% Hy-Soy agar square petri dish before incubation for 18–24 h at 37°C in a humidified chamber. Each plate was spiked with duplicate samples of bacteria incubated in pre-immune or post-vaccination sera taken after the 4^th^ dose, and normalized to a control sample of bacteria incubated in PBS buffer alone. The diameter of motility was captured with a ChemiDocMP system (Biorad, CA) and measured using Image Lab Version 5.1 (BioRad, CA).

### Flow cytometry analysis for antibody binding to target strains

KP bacterial cultures grown 12–18 hours at 37°C / 250 rpm in HS media were washed with PBS and the OD_600nm_ adjusted to 0.8 from which 100 μL were centrifuged 10 minutes at 4°C, 15,000 *x g*. The bacterial pellet was then suspended in 100 μL of mouse immune serum diluted in the flow cytometry staining buffer (1 x PBS, 1% fetal bovine serum [Gemini Bioproducts, CA], 0.02% sodium azide). The samples were incubated for 1 hour at 4°C, then washed three times with the staining buffer followed by incubation for 1 hour at 4°C with FITC labeled goat anti-mouse IgG (BioLegend, CA) at 9 ng/μL. Stained bacteria samples were acquired on a LSR-II (custom built) system (BD, San Jose, CA). FSC and SSC were set to logarithmic amplification. The data were analyzed using the software package FlowJo V10 (Ashland, OR).

### Statistical analysis

All statistical analyses were performed with GraphPad Prism v6.0 (GraphPad Software Inc., CA). Survival analyses for Kaplan-Meier curves were accomplished by log-rank test. Fifty percent lethal dose (LD_50_) values were calculated by regression analysis. Comparisons between differences in motility inhibition were assessed by two-tailed Student’s t-test. Statistical significance for ELISA titers, recovered CFU after bacterial challenge, and OPA uptake were assessed by two-tailed Mann-Whitney. P-values below 0.05 were considered as a significant difference between groups.

## Results

### Genetic engineering of KP reagents strains to produce conjugate vaccine OPS

**Fig 1:**
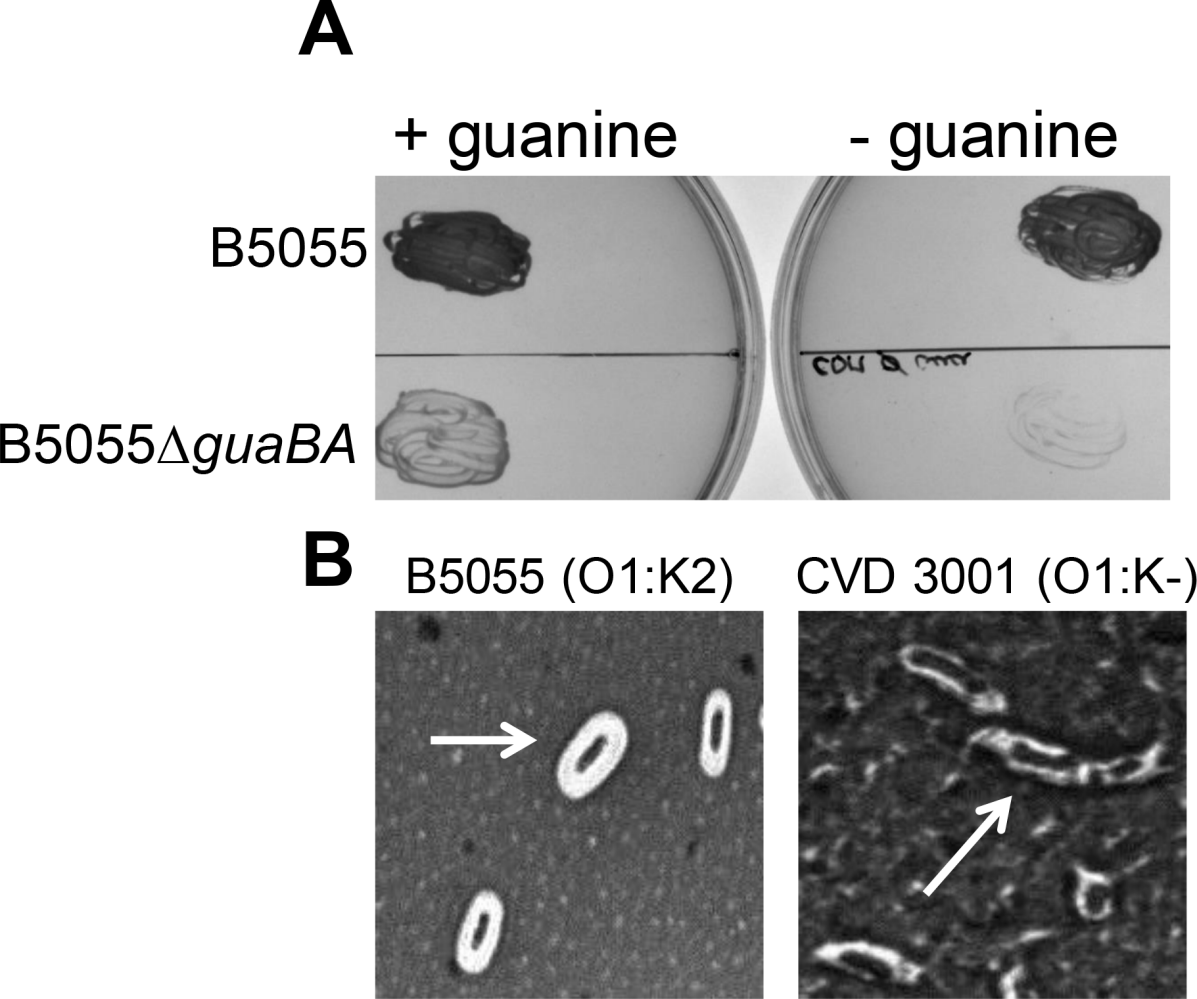
Characterization of KP O1 reagent strain for OPS purification. (A) Growth of wild-type B5055 and B5055 Δ*guaBA* mutant on chemically defined agar medium with (+) or without (-) 0.01% guanine. (B) India ink staining of the polysaccharide capsule of B5055 and CVD 3001 (B5055 Δ*guaBA* Δ*wzabc*). Arrow indicates bacterial cells.

KP B5055 (O1:K2) was used to engineer reagent strain CVD 3001, by deleting the genes for *guaBA* (guanosine biosynthesis, attenuating) and *wzabc* (removes capsule, attenuating and simplifies OPS purification). The mutant was compared to wild-type phenotypically by measuring guanine auxotrophy, lack of capsule expression and attenuation. Whereas wild-type B5055 grew on CDM lacking guanine (Fig 1A), and displayed a thick white halo after capsule staining (Fig 1B), deletion of *guaBA* abolished growth in the absence of exogenous guanine and further deletion of the capsule genes rendered the strain non-encapsulated as seen by the absence of the white halo surrounding the cell. CVD 3001 was highly attenuated in the mouse model of infection with an IP LD50 > 10^9^ CFU compared to ~ 10^4^ CFU for B5055. A similar strategy was utilized to generate reagent strain CVD 3002, by deletion of the *wzabc* genes from KP 4425/51 (O5:K57). KP 7380 is a non-encapsulated O2 isolate, and hence was used as a reagent strain without any further modification. KP 390 (O3:K11) was found to be refractory to our attempts at genetic engineering. This strain was thus used as a reagent strain in its native form.

### Production and characterization of KP OPS and PA flagellin proteins

**Fig 2:**
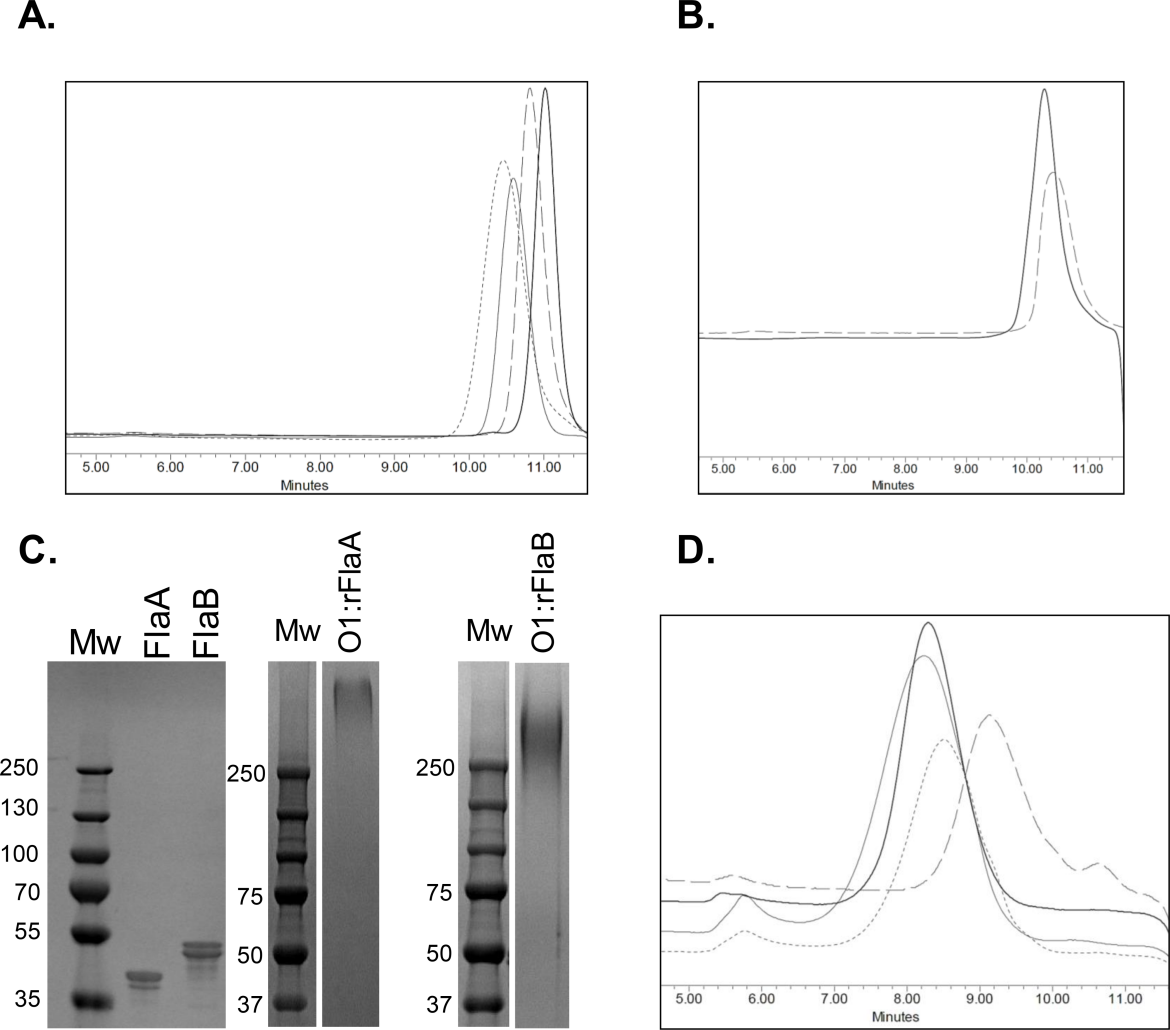
Biochemical and biophysical characteristics of purified KP OPS, PA flagellin antigens and conjugates. (A) HPLC-SEC with RI detection for purified KP O1 (short dash trace), O2 (long dash trace), O3 (thin black trace) and O5 (thick black trace) OPS molecules. (B) HPLC-SEC for rFlaA (dash trace) and rFlaB (solid trace) proteins. (C) SDS-PAGE and Coomassie stain of rFlaA or rFlaB, alone or conjugated to KP O1 OPS (O1:rFlaA and O1:rFlaB). Mw = molecular weight marker. (D) HPLC-SEC for O1:rFlaB (thin black trace), O2:rFlaA (long dash trace), O3:rFlaB (short dash trace), O5:rFlaA (thick black trace).

KP OPS was obtained from fermentation cultures of KP reagent strains, and purified by a scalable manufacturing approach to yield large amounts of highly purified polysaccharide with low residual protein (<1%) and endotoxin (<0.01 endotoxin units/μg). HPLC-SEC analyses of the purified polysaccharides revealed that they were each comprised of a relatively homogenous molecular size population that was similar among the 4 OPS types (Fig 2A). Monosaccharide composition analyses by HPAEC-PAD for depolymerized KP OPS further confirmed that KP O1 and O2 were polygalactans whereas KP O3 and O5 were comprised of mannose (not shown). Purified PA FlaA1 and FlaB, expressed as recombinant proteins in *E*. *coli*, generated protein bands at the predicted molecular weight based on amino acid sequence and defined peaks by HPLC-SEC congruent with the monomeric form (Fig 2B and 2C), with less than 0.1 endotoxin units/μg protein. KP OPS molecules were linked at the reducing end with an aminoooxy-thiol linker to PA Fla protein lysines that had been derivatized with a maleimide molecule. Conjugation by this approach generated sun-type neoglycoconjugates for which the OPS chain remains intact, and extends from the protein surface (S1 Fig). HPLC-SEC analysis of the purified glycoconjugates indicated that each maintained a relatively narrow size distribution that was similar among the different conjugates (Fig 2D), and with roughly equivalent polysaccharide to protein ratios (Table 2). Further we found that whereas rFlaA and rFlaB strongly activated TLR5 signaling, conjugation reliably ablated TLR5 bioactivity (S2 Fig).

**Table 2:** Polysaccharide (PS) to protein ratios of conjugates used in this study.

Conjugate	PS:Protein	Experiment for which the conjugate was used
**O1:FlaA**	0.90	Mouse immunization
**O1:FlaB**	0.21	
**O1:FlaB**	0.55	Rabbit immunization
**O2:FlaA**	0.28	
**O3:FlaB**	0.40	
**O5:FlaA**	0.26	

### Humoral immune response to monovalent KP-O1 conjugates with PA rFlaA or rFlaB

**Fig 3:**
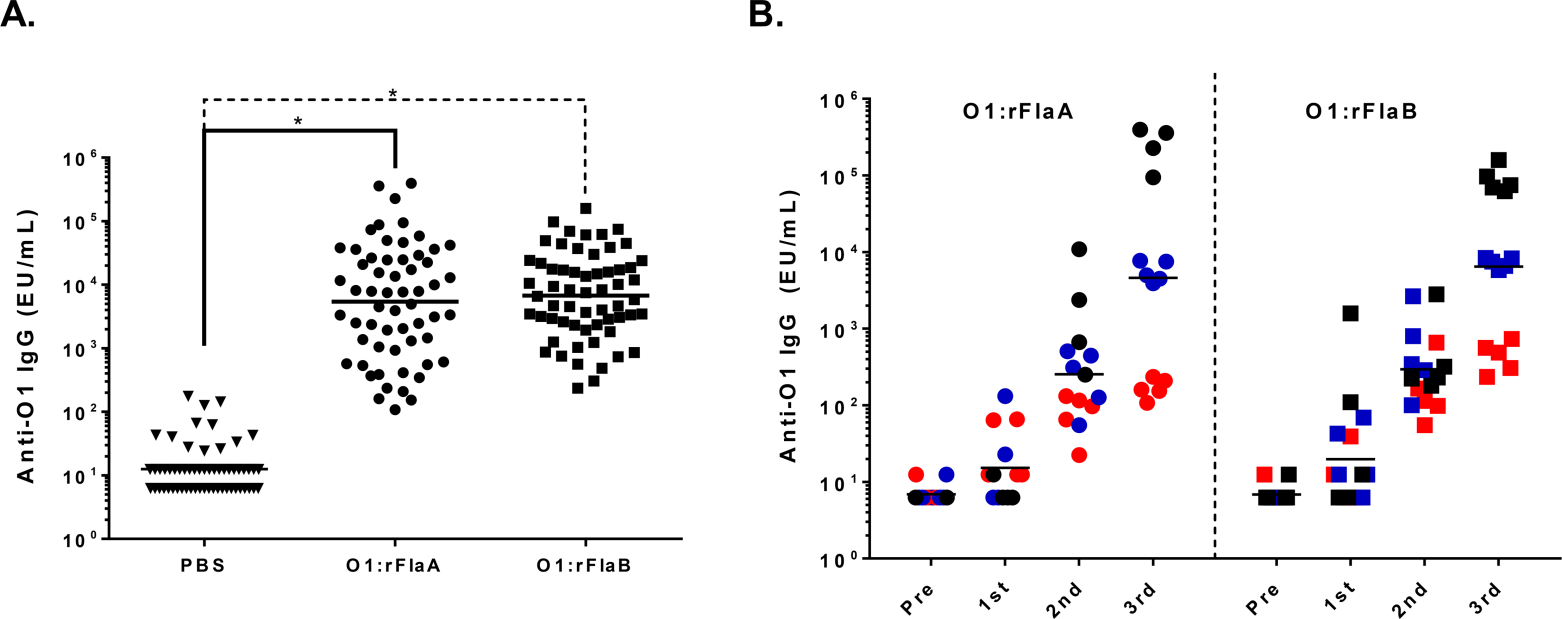
Immunogenicity and kinetics of anti-OPS IgG induced by monovalent KPO1 conjugates with PA rFlaA or rFlaB in mice. (A) Anti-OPS IgG was assessed in sera obtained 28 days after the last dose from mice (n = 59–60) immunized with three doses of PBS (triangles), O1:rFlaA (circles) or O1:rFlaB (squares). Statistical significance by Mann-Whitney is indicated. * P ≤ 0.0001. Each point indicates the serum titer from an individual mouse. Bars indicate the geometric mean titer. (B) Anti-OPS IgG titers in select high (black, n = 4–5), moderate (blue, n = 5) or low (red, n = 5) responders in sera taken directly prior to immunization or 14 days after the 1^st^, 2^nd^, or 3^rd^ dose of O1:rFlaA (circles) or O1:rFlaB (squares). Bars indicate the geometric mean titer.

**Fig 4:**
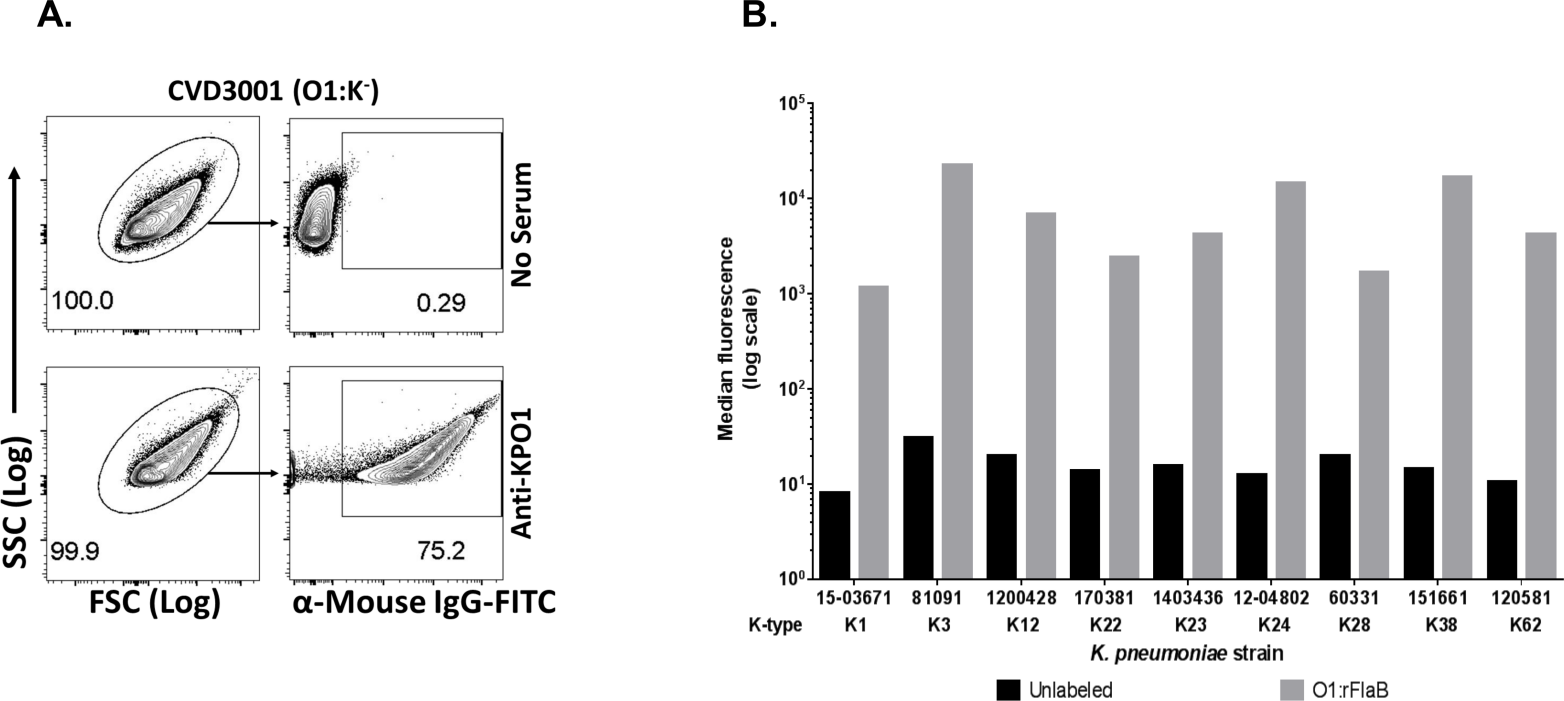
Binding by O1 conjugate induced antibodies to KP O1 OPS isolates expressing different capsule types. Sera from mice immunized with O1:rFlaB (anti-KPO1) were incubated with different KP isolates and binding was assessed by flow cytometry using fluorescently labeled (FITC) secondary detection antibody. KP incubated with FITC labeled KP alone (in the absence of serum) was used as control. (A) Forward scatter (FSC)/ side scatter (SSC) plots are shown for CVD 3001 as a representative for the group, indicating the selected population for analysis (left), and SSC/ FITC analyses of CVD 3001. Selected population percentages are indicated. (B) A panel of KP O1 clinical isolates from UMMC were assessed as above. The mean fluorescence intensity for bacteria labeled with conjugate sera (O1:rFlaB) over background (Unlabeled) is indicated.

In order to compare the immune response to KP O1 OPS when conjugated to PA rFlaA and rFlaB, mice were immunized IM with monovalent conjugates of O1:rFlaA or O1:rFlaB or PBS (placebo). We found that the conjugates induced comparably robust anti-OPS IgG levels with similar end point titers and levels of seroconversion in sera obtained after three doses (Fig 3A). Analyses of the kinetics of antibody induction in a subset of vaccinated animals revealed negligible anti-O1 IgG levels after the priming dose, progressively higher antibody levels after the 1^st^ and 2^nd^ booster doses and a pattern of high, moderate or lower endpoint titers after three doses, (Fig 3B). To ascertain whether conjugate-induced anti-OPS antibodies maintained the ability to bind encapsulated O1 OPS bacteria in the context of different capsule types, flow cytometry analyses were conducted with pooled sera from mice immunized with O1:rFlaB. We assessed binding to a panel of KP O1 clinical isolates of various capsule types. As is shown in Fig 4, robust binding by the immune sera was seen for all of the strains irrespective of capsule type, in a manner comparable to that found for unencapsulated KPO1 reagent strain CVD 3001.

### Rabbit immunization with quadrivalent conjugate or unconjugated antigens

**Fig 5:**
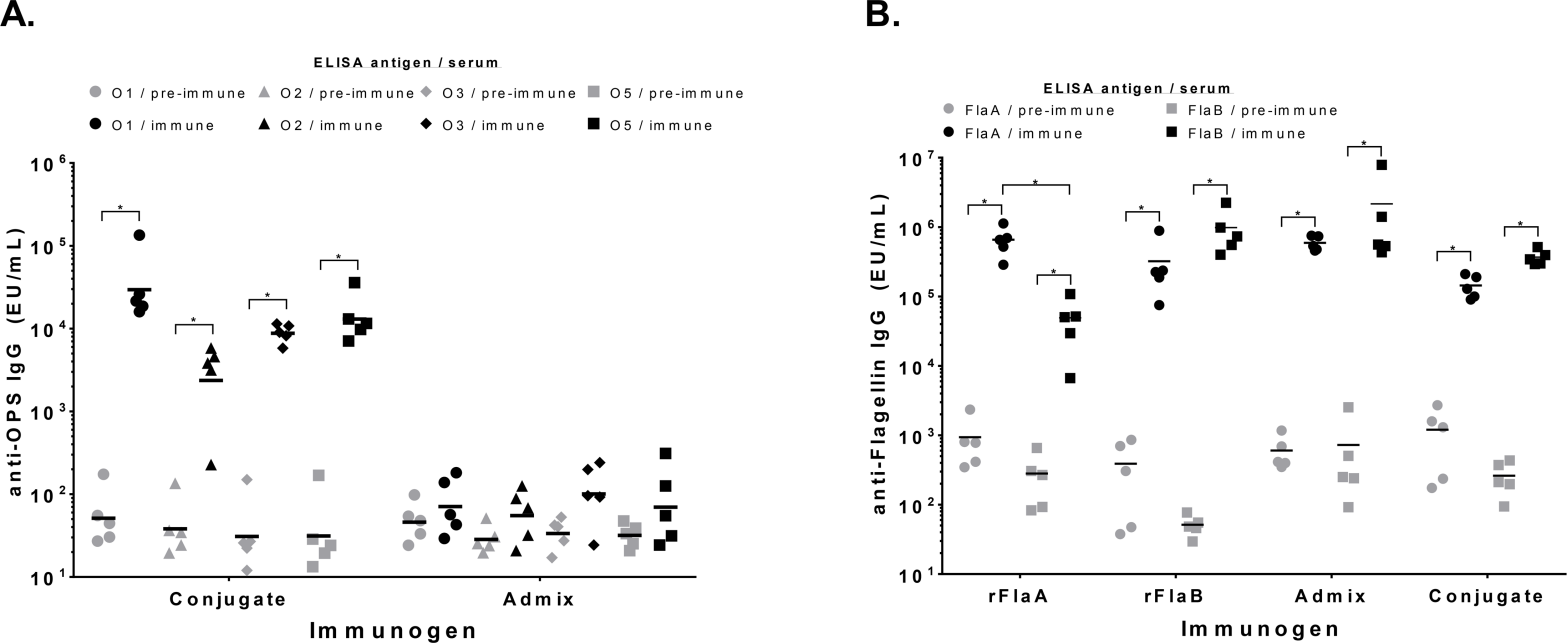
Immune response in rabbits to immunization with conjugates or admixed conjugate vaccine components. (A) Anti-polysaccharide IgG titers for O1 (circles), O2 (triangles), O3 (diamonds) or O5 (squares) OPS were assessed by ELISA for sera obtained from rabbits prior to immunization (pre-immune, grey symbols) and 14 days after the third dose (immune, black symbols) with the conjugate formulation or admixed OPS and flagellin components. (B) IgG antibody levels for rFlaA (circles) or rFlaB (squares) were assessed in sera obtained prior to vaccination (pre-immune, grey symbols) or 14 days after the third dose (immune, black symbols) of conjugate, admixed components or individual flagellin proteins. Each points represents an individual rabbit. Bars indicate the geometric mean titer. Statistical significance assessed by Mann-Whitney is indicated. *P < 0.05.

Since anti-polysaccharide IgG titers were similar when O1 OPS was conjugated to either rFlaA or rFlaB, a four component vaccine formulation was generated by conjugation of the four KP OPS types with either PA rFlaA or rFlaB proteins (O1:rFlaB + O2:rFlaA + O3:rFlaB + O5:rFlaA). The four component conjugate formulation was assessed for immunogenicity in rabbits, measuring induction of serum IgG to the six vaccine antigens prior to immunization and after three vaccine doses. As comparators to assess the response to these antigens when present in unconjugated form, separate groups of rabbits were immunized with admixed purified OPS and flagellin components (O1 + O2 + O3 + O5 + rFlaA + rFlaB), or individual flagellin proteins alone. Rabbits immunized with the conjugate formulation generated maximal anti-OPS IgG levels after three doses that were comparably high for all four OPS types (Fig 5A, S3 Fig). In contrast, immunization with unconjugated admixed vaccine components failed to induce significant anti-OPS IgG titers. High antibody levels against both of the PA flagellin proteins were also seen in rabbits immunized with the four-component conjugate formulation, however, the titers were lower in overall magnitude relative to those obtained with admixed components or the individual flagellin proteins (Fig 5B). Analyses of cross-reactive antibodies in sera from rabbits immunized with individual flagellin proteins indicated that the serum antibody response was generally higher for the homologous flagellin type compared to the heterologous species, suggesting production of antibodies against the serotype specific flagellin epitopes.

### Passive transfer protection with rabbit quadrivalent conjugate antisera against KP and PA infection

**Fig 6:**
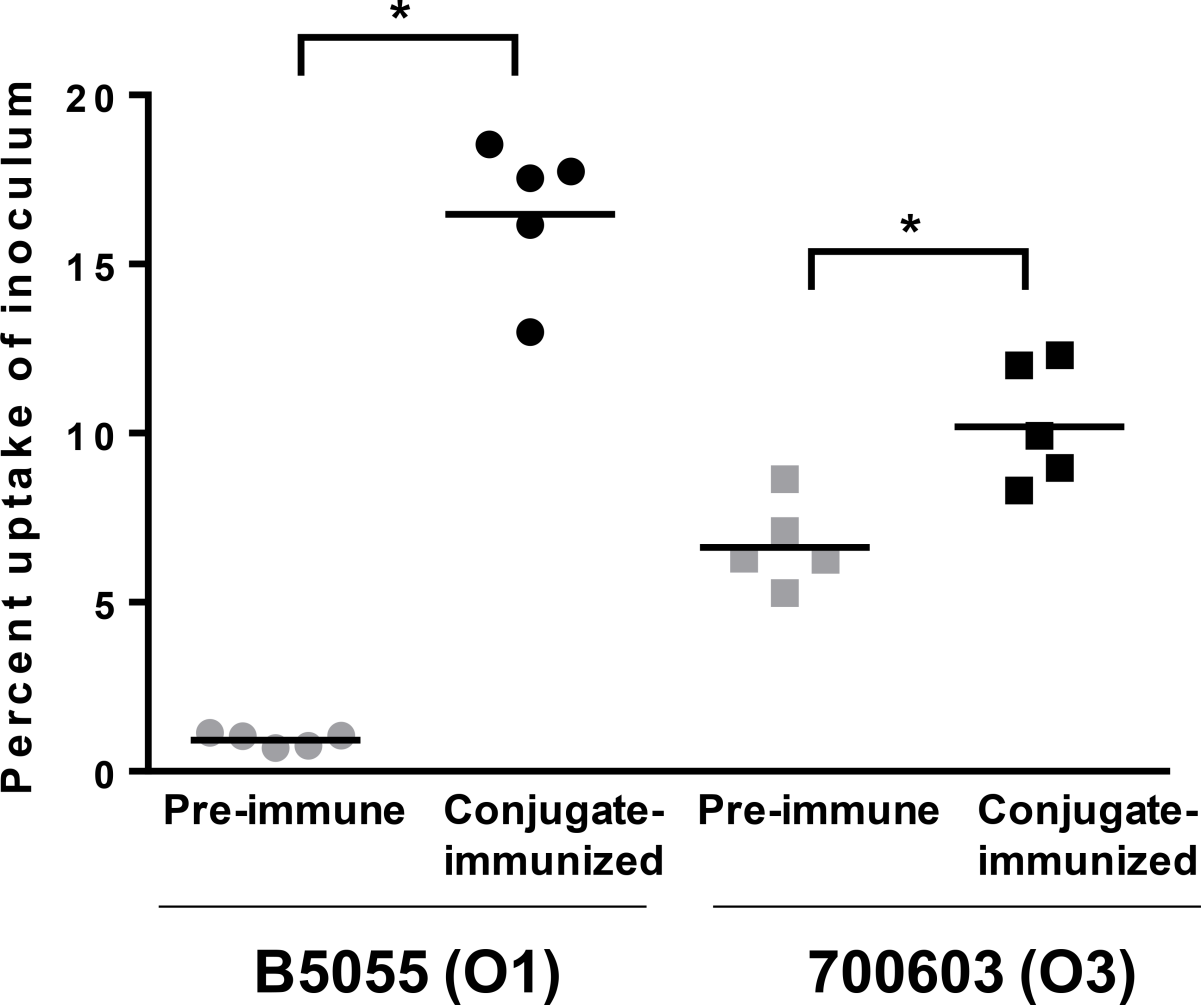
Opsonophagocytic uptake of KP with sera from conjugate-immunized rabbits. The percent uptake of KP B5055 (O1, circles) or 700603 (O3, squares) inoculum into J774 macrophages was assessed after incubation with pre-immune (grey symbols) or conjugate-induced (black symbols) sera from individual rabbits. P value by Mann-Whitney for pre-immune compared to immune sera is indicated. * P ≤ 0.05. Results are the average of duplicate wells for each serum sample and are representative of two independent experiments.

Monoclonal antibodies against KP OPS have previously been shown to promote uptake into macrophages by opsonophagocytosis [16]. To determine whether conjugate induced anti-OPS antibodies would also mediate OPA, uptake of KP O1 (B5055) or KP O3 (700603) bacteria into J774 mouse macrophages was assessed after opsononization with paired pre-immune or conjugate-induced sera from individual rabbits. We found that whereas pre-immune sera promoted negligible uptake, conjugate-induced sera promoted significant OPA activity (Fig 6).

**Fig 7:**
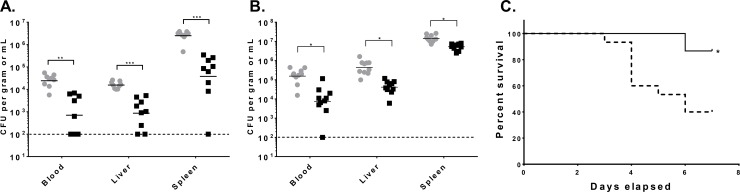
Protective efficacy of passively transferred conjugate-induced antibodies against systemic KP infection in mice. (A,B) Naïve mice were passively administered IP pooled pre-immune (grey circles) or conjugate-induced (black squares) antisera followed by intravenous infection with KP O1 strain B5055 (n = 9/group) (A), or KP O3 strain 700603 (n = 10/group) (B). Mice were euthanized 4 hours after inoculation, and viable colony counts were determine in the blood, liver and spleen as indicated. Statistical significance for post-immune compared to pre- immune sera by Mann-Whitney are shown. Each point represents an individual mouse. Bar = geometric mean. (C) Naïve mice (15/group) were passively administered pre-immune (dash line) or conjugate-induced (solid line) rabbit sera and then assessed for mortality after intravenous infection with KP B5055. Results are the combination of two independent experiments. P value by log-rank test for pre-immune compared to immune sera is indicated. * P ≤ 0.05, ** P≤ 0.001, *** P ≤ 0.0001.

To further investigate whether the vaccine-induced anti-KP OPS antibodies would promote bacterial clearance *in vivo* and offer protection against infection, we passively administered pooled pre-immune or conjugate-induced rabbit sera to mice and assessed bacterial clearance in blood, liver and spleen tissues after IV infection with encapsulated KP B5055 (O1 OPS type) or 700603 (O3 OPS type) isolates. Strikingly, we found that transfer of immune sera markedly reduced the level of KP organisms in a manner that was comparable for both the KPO1 and KPO3 isolates (Fig 7A and 7B). Reduction in KP CFU also correlated with protection against mortality, wherein mice administered conjugate immune sera were significantly protected against IV infection with B5055 (Fig 7C).

**Fig 8:**
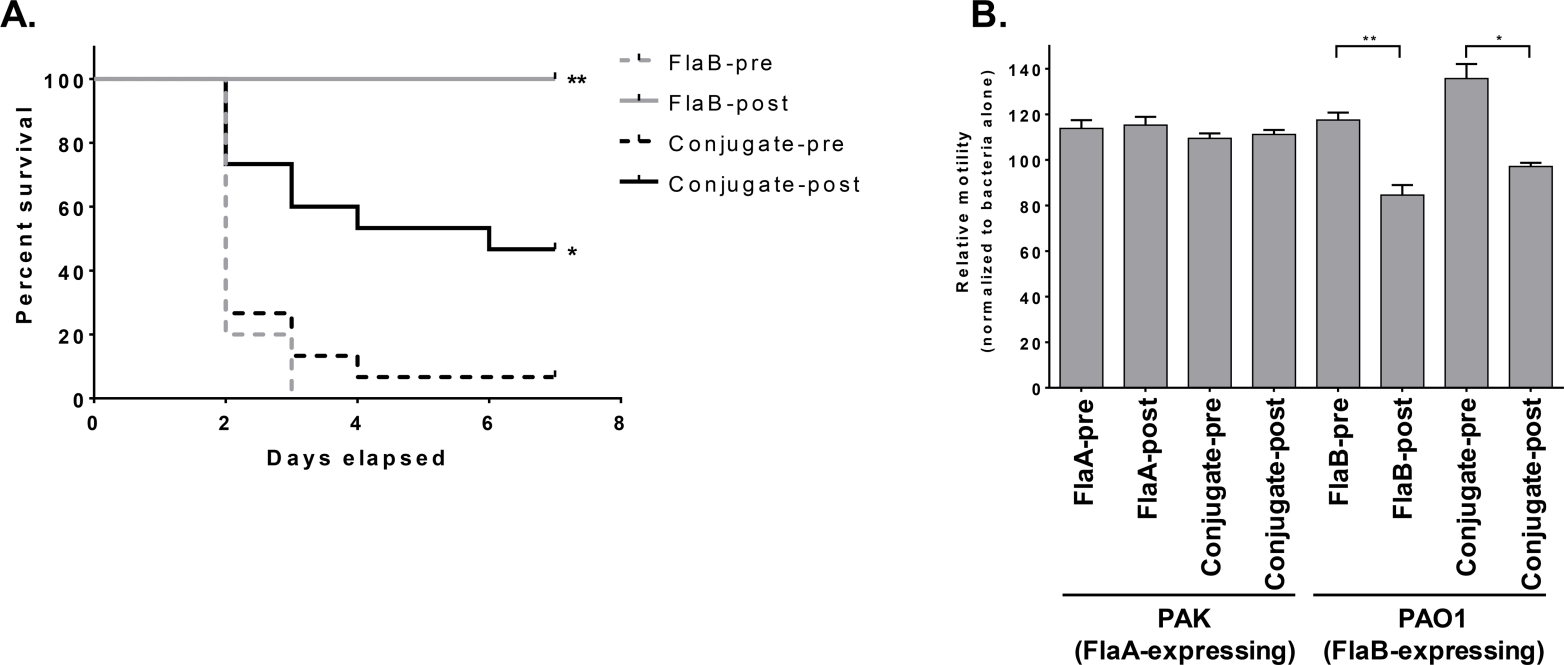
Functional activity of anti-PA Fla antibodies *in-vitro* and *in-vivo*. (A) Naïve mice (15 per group) were passively transferred with pooled paired pre-immune (dashed line) or vaccine-induced (solid line) antisera from rabbits immunized with the conjugate (black line) or rFlaB alone (grey line) and then infected by burn wound inoculation with PA strain M2. Results are the combination of two independent experiments. P value by log rank assay for immunized mice and immune sera relative to control is indicated. (B) Pooled pre-immune or immune sera from rabbits immunized with FlaA, FlaB or the 4-component conjugate were assessed for motility inhibition with PA strains PAK (FlaA) or PAO1 (FlaB). Results are from duplicate samples on three separate plates and are normalized to motility with PBS alone and are representative of two independent experiments. Mean +/- standard error are shown. P value by paired t-test is indicated. * P≤ 0.05, ** P ≤ 0.001.

To assess whether the conjugate induced antisera would also protect against PA infection, mice were passively administered either pre-immune sera or paired immune sera from conjugate or rFlaB-only immunized rabbits and then infected with FlaB PA strain M2 in a burn-wound infection model (Fig 8A). Whereas mice administered pre-immune sera all succumbed to infection by day 3, all animals receiving the FlaB immune sera survived. Mice receiving the conjugate sera were also significantly protected, although at a lower level compared to the FlaB immune sera. As controls for the burn wound procedure, a separate group of bystander mice that were burned but not infected all survived (not shown). By comparison, transfer of antisera to unconjugated or conjugated rFlaA failed to protect mice against burn wound infection with FlaA PA strain PAK (not shown). We then determined whether conjugate-induced anti-flagellin antibodies could mediate functional motility inhibition for PA. For this, we compared sera from rabbits immunized with the individual flagellin proteins or the 4-component conjugate with representative FlaA (PAK) or FlaB (PAO1) strains. Whereas conjugate and anti-FlaB sera could inhibit PAO1 motility, sera from rabbits immunized with unconjugated FlaA or conjugated to OPS did not inhibit PAK motility (Fig 8B).

## Discussion

Antimicrobial resistance is a major problem globally, forecasted to severely impact public health with increasing rates of mortality due to AMR infections [37]. With the paucity of new antibiotics and novel drug classes in the pipeline, alternative and adjunct strategies are urgently needed to prevent poorly or otherwise unmanageable AMR bacterial infections [38]. Immunoprophylactic approaches are not affected by the molecular mechanisms of resistance inherent in AMR, and can protect against infection with antibiotic susceptible and resistant strains. Indeed, there is convincing prior evidence for the promise of vaccines in counteracting infections with AMR pathogens. The emergence in the late 1990s of AMR *Haemophilus influenzae B* (Hib) and *S*. *pneumoniae* isolates coincided fortuitously with the introduction of the newly developed glycoconjugate vaccines for these pathogens. Surveillance studies conducted prior to and following vaccine introduction documented that the use of these vaccines led to a marked reduction of the overall incidence of Hib and pneumococcal disease that included AMR strains [4, 39]. Vaccine approaches offer the additional benefit in lowering the necessity for antibiotic use, which is an independent contributing factor for acquisition of AMR phenotypes [3]. There are a multitude of studies centered on the development of PA vaccines that have assessed a range of antigens including outer membrane proteins, O polysaccharides, exopolysaccharides, toxins, pili and flagella [5]. However, comparatively less effort has been directed towards development of a vaccine for KP [9]. Among these, even fewer have addressed the notion of targeting both pathogens simultaneously. Early phase clinical trials assessed co-administration of separate multivalent KP and PA vaccine preparations comprised of 24 different KP capsule types or 8 different PA OPS types conjugated to PA exotoxin A. Unfortunately, marked variability in the immune response to individual PS components was noted [40]. KP OPS-based vaccines require fewer vaccine antigens to achieve broad coverage, yet there is a paucity of published studies that have evaluated OPS glycoconjugates. Several studies have reported the development of glycoconjugate vaccines with KP O1 OPS, including as conjugates with KP iron regulated outer membrane proteins and tetanus toxoid [41, 42]. The recent reports of protective monoclonal antibodies directed against KP OPS further support the utility of targeting the KP O antigen [43–45]. To our knowledge this is the first study to test an OPS vaccine formulation that includes all four of the predominant O types.

We found that conjugation to a protein carrier significantly enhanced anti-OPS immunity relative to immunization with the unconjugated polysaccharide antigen. Polysaccharides are generally thymus-independent antigens that are poorly immunogenic and do not induce immunologic memory, class-switch, or affinity maturation. Covalent coupling of bacterial polysaccharides with proteins allows for T-cell help, and has enhanced the magnitude, quality and duration of the humoral immune response [46]. Heterologous pathogen carrier proteins are used widely in commercially available glycoconjugates including the aforementioned Hib and pneumococcus vaccines [47]. The most commonly used carrier proteins in these vaccines are tetanus toxoid or inactivated derivatives of diphtheria toxoid. However, these antigens are already administered during routine immunization schedules and high levels of preexisting anti-carrier immunity have been associated with diminished immunogenicity through epitopic suppression mechanisms [48]. The use of a novel, pathogen-relevant carrier protein offers the potential to extend valency or coverage for other pathogens. The sole available vaccine that utilizes this strategy is the pneumococcal conjugate vaccine Synflorix (GlaxoSmithKline) that uses protein D from non-typeable *Haemophilus influenzae* (NTHi) as a carrier with intent of extending coverage to prevention of NTHi otitis media [49]. Work from our group and others has found that flagellin proteins (e.g., from *Salmonella* and *Burkholderia*) are effective carrier proteins when linked to the homologous pathogen OPS molecules [19, 32, 50, 51]. PA flagellin proteins have been reported previously as effective carriers for PA alginate exopolysaccharide in murine studies where measurable antibody levels were induced to both carrier and hapten antigens and protection was imparted against PA pneumonia infection [50]. Our results here indicate that PA flagellin is also an effective carrier for KP OPS, and furthermore that rFlaA and rFlaB maintain equivalent carrier function. While anti-flagellin titers were lower upon conjugation, anti-flagellin antibodies induced after conjugate vaccine immunization demonstrated functional motility inhibition, and protected in mouse models of burn wound infection with a prototype FlaB isolate. We found, however, that anti-rFlaA antibodies induced by conjugated or unconjugated protein offered negligible protection against burn wound infections and did not inhibit motility with PAK (FlaA1). This result is unexpected, as recombinant FlaA has generated functional antibodies in prior studies [52, 53]. Both PA FlaA and FlaB proteins can become glycosylated at two sites in the serotype specific variable region [54, 55]. The carbohydrate moieties associated with FlaA are larger (6–11 saccharides) than those associated with FlaB (disaccharide). Our recombinant flagellin proteins expressed in *E*. *coli* do not contain these modifications. It is conceivable that the longer carbohydrate modifications on FlaA impede accessibility of antibodies to the protein surface, in a manner that is less prominent for FlaB. It is also possible that the recombinant FlaA protein was improperly folded resulting in disruption of key protective epitopes. However, as rFlaA maintained TLR5 bioactivity, this suggests that the protein is at least partially correctly folded. Alternatively, it is further conceivable that our PAK target strain may have downregulated flagellin *in vivo* as a consequence of alginate production, a known biofilm promoting factor that is expressed during PA burn wound infections [56, 57].

We assessed herein protection against challenge by distinct routes for KP and PA. Future work should assess protection in additional challenge models, including those for pneumonia that is a common complication for KP and PA nosocomial infections [1]. PA flagellin-based vaccines have been assessed in clinical studies, where they were found to be safe and immunogenic [58]. Intriguingly, parenteral immunization with PA Fla proteins induced IgG antibodies that could be found in the sera as well as the pulmonary mucosa [59]. Effective immunity at the mucosal surface is desirable as KP and PA can cause bacterial pneumonias. They can also asymptomatically colonize the gastrointestinal tract and nasopharynx of susceptible individuals [60, 61]. Genomic sequencing of *Klebsiella* isolates from septic patients has indicated that the infecting strain in most (>80%) instances is genetically identical to the colonizing isolate, suggesting that the colonizing strain is a reservoir for infection [60]. Future studies should thus also address whether the quadrivalent KP-OPS:PA-Fla vaccine can prevent bacterial colonization at mucosal surfaces, as has been documented for pneumococcal conjugate vaccines [62]. Additionally, whereas KP infections have generally been associated with impaired immunologic function consequent to age or disease comorbidity and in hospital settings, community acquired highly mucoid hypervirulent KP (hvKP) strains, first described in the Asia Pacific rim, have been associated with septic and focal infections including liver abscesses in otherwise healthy individuals [63]. Mouse pathogenicity analyses of a prototype hvKP K1 isolate indicated that the O antigen was an essential virulence factor [64]. It would thus also be of importance to determine in the course of future studies whether anti-OPS antibodies maintain protection against these highly mucoid isolates [15, 17, 43, 65].

## Supporting information

S1 Fig Molecular architecture of the sun-type glycoconjugates generated for this study. (TIF)

S2 Fig Reduction in PA flagellin mediated TLR5 bioactivity after conjugation to KP OPS. TLR5 activity of (A) rFlaA or (B) rFlaB for flagellin proteins alone (diamonds) or as conjugates with O1 OPS (circles).
(TIF)

S3 Fig Geometric mean IgG titers to OPS and flagellin components prior to immunization or after 3 or 4 doses. (A) Anti-OPS IgG titers to KP O1, O2, O3 and O5 prior to and after 3 or 4 doses of conjugated or admixed vaccine antigens. (B) Anti-flagellin IgG titers prior to and after 3 or 4 doses of conjugate, rFlaA, rFlaB or admixed vaccine antigens. Mean +/- standard error are shown. Statistical significance determined by 2-tailed unpaired Mann-Whitney test. *P ≤ 0.05.
(TIF)

S1 Table Primers used in this study. (DOCX)

S2 Table Sequences of the codon optimized a-type and b-type flagellin cloned in pTrcHis—TOPO by TA cloning and produced as recombinant proteins in *E*. *coli* BL21. (DOCX)
