# Supplementary figures and images for: Development of a broad spectrum glycoconjugate vaccine to prevent wound and disseminated infections with Klebsiella pneumoniae and Pseudomonas aeruginosa
Source: PLoS One. 2018 Sep 6;13(9):e0203143. doi: 10.1371/journal.pone.0203143 (PMC6126813; doi:10.1371/journal.pone.0203143)

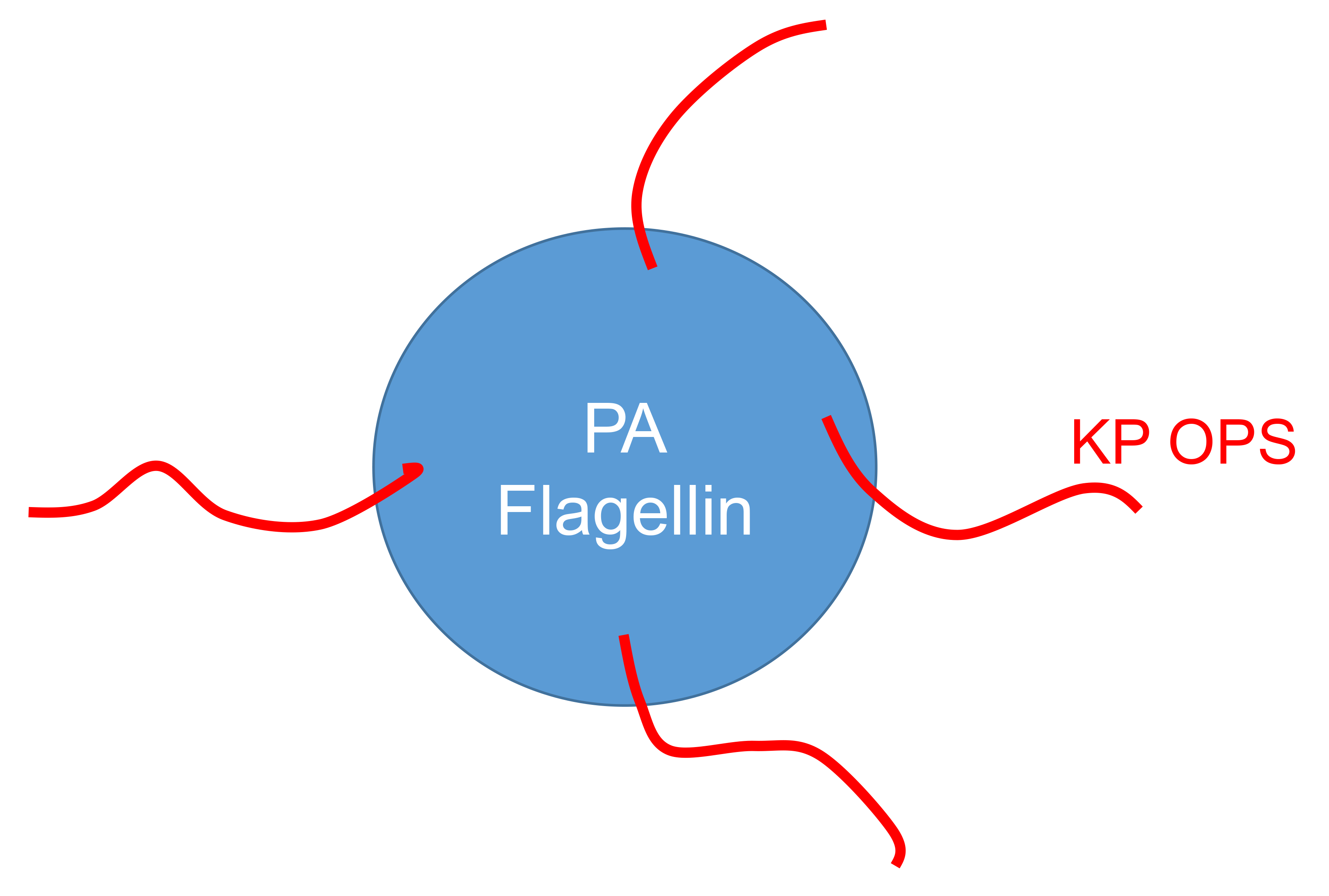

Supplement: S1 Fig — (TIF) [file pone.0203143.s001.tif]

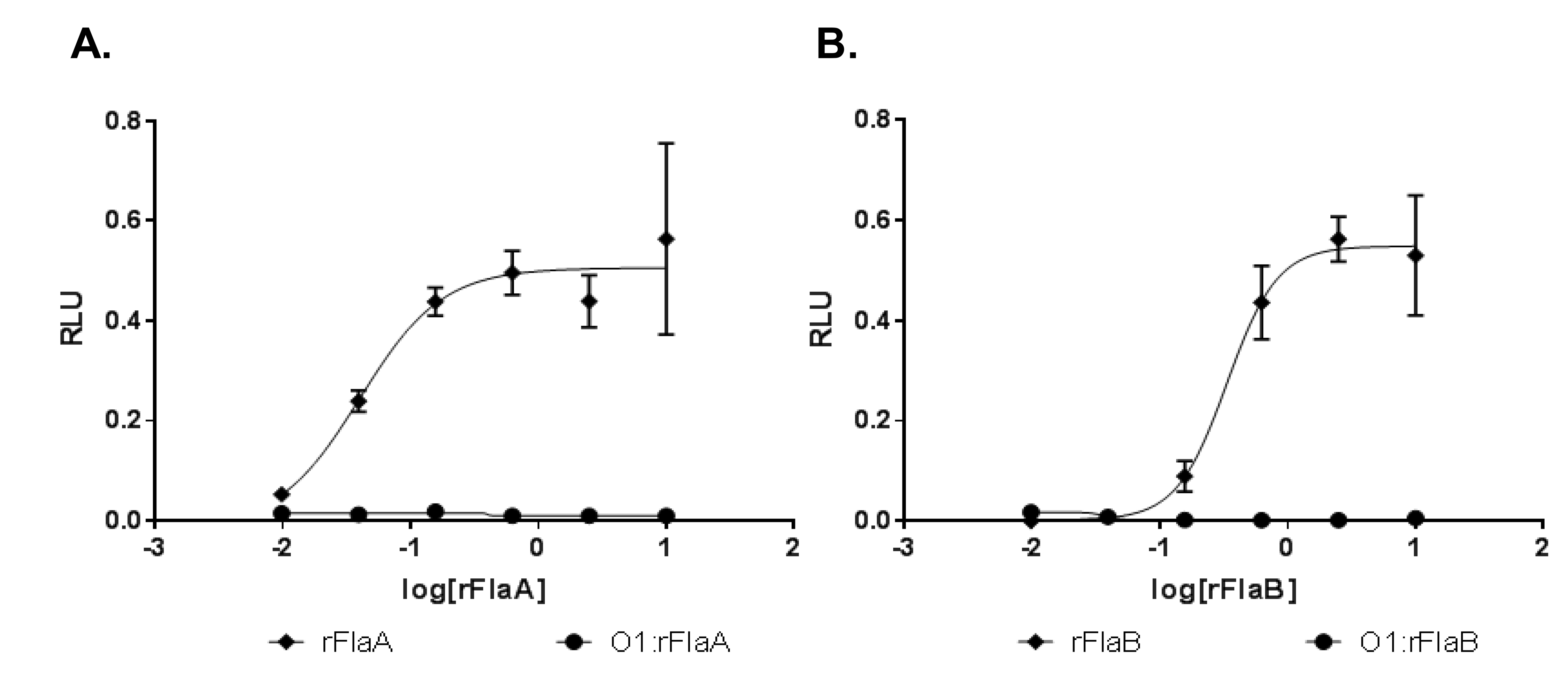

Supplement: S2 Fig — TLR5 activity of (A) rFlaA or (B) rFlaB for flagellin proteins alone (diamonds) or as conjugates with O1 OPS (circles). (TIF) [file pone.0203143.s002.tif]

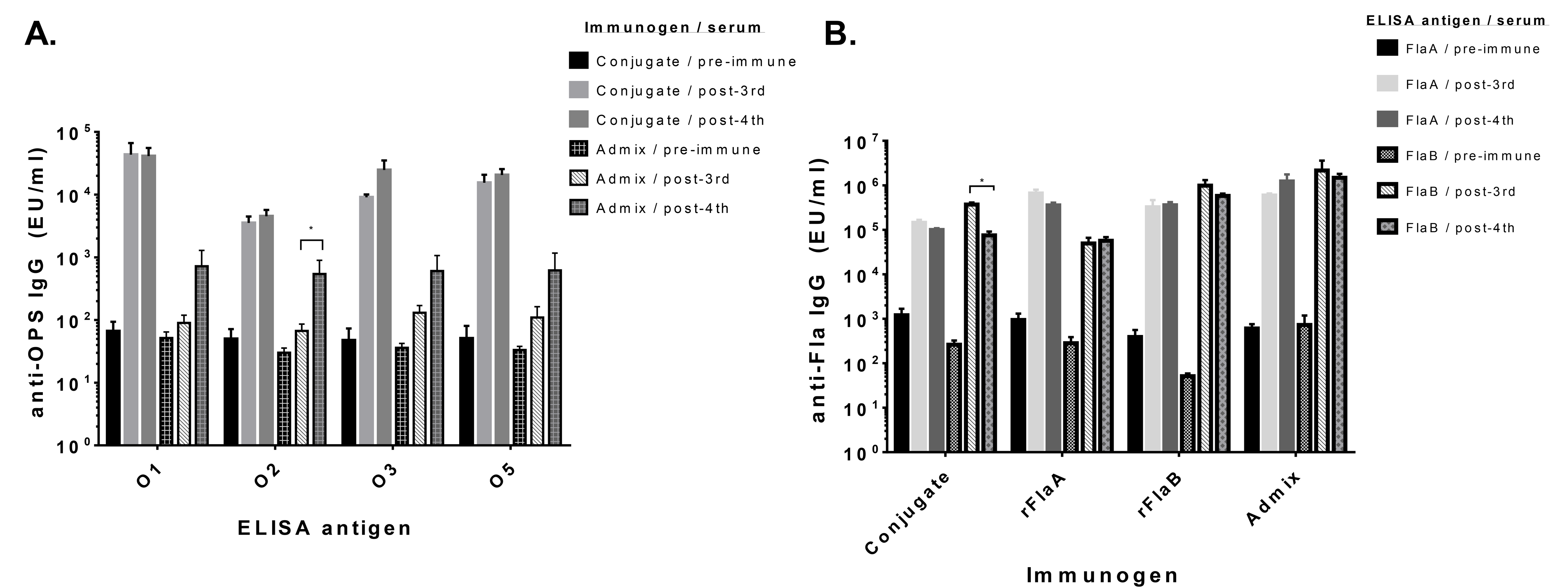

Supplement: S3 Fig — (A) Anti-OPS IgG titers to KP O1, O2, O3 and O5 prior to and after 3 or 4 doses of conjugated or admixed vaccine antigens. (B) Anti-flagellin IgG titers prior to and after 3 or 4 doses of conjugate, rFlaA, rFlaB or admixed vaccine antigens. Mean +/- standard error are shown. Statistical significance determined by 2-tailed unpaired Mann-Whitney test. *P ≤ 0.05. (TIF) [file pone.0203143.s003.tif]
