# Supplementary material for: Development of a broad spectrum glycoconjugate vaccine to prevent wound and disseminated infections with Klebsiella pneumoniae and Pseudomonas aeruginosa
Source: PLoS One. 2018 Sep 6;13(9):e0203143. doi: 10.1371/journal.pone.0203143 (PMC6126813; doi:10.1371/journal.pone.0203143)
Supplement: S1 Table — (DOCX) [file pone.0203143.s004.docx]

| **Name** | **Target** | **5’ -> 3’ sequence** |
| --- | --- | --- |
| guamut_F | Kp upstream flanking region of *guaBA* | ***GGTCGACGGATCCCCGGAAT***GGAGTAATCCCCGGCGTTAG |
| guaBA_R |  | TGATTGGTCTGACTGGACGC |
| guaBA_F | Kp downstream flanking region of *guaBA* | GGGTAGATGATCACCGGCAG |
| guamut_R |  | ***GAAGCAGCTCCAGCCTACAC***GGGCAATATCTCGACCAGGG |
| wza_F | Kp upstream flanking region of K2 *wza* | GAGCCGACTCTAGGGTGGC |
| wzamut_R |  | ***GAAGCAGCTCCAGCCTACAC***TAATGTCACATCATCAGTAAATCAAAATTTG |
| K2_wzcmut_F | Kp downstream flanking region of K2 *wzc* | ***GGTCGACGGATCCCCGGAAT***GTAATAGATATGTTATAGAGTTTGGAGGGGAG |
| K2_wzc_R |  | TATTTAATTTCCCTCTTTCATCCTGTAATGTT |
| P1 | pKD13 kanamycin resistant cassette | GTGTAGGCTGGAGCTGCTTC |
| P4 |  | ATTCCGGGGATCCGTCGACC |
| fla_F | Codon optimized a-type and b-type flagellin | ATGGCCCTGACGGTGAATAC |
| fla_R |  | TTAACGCAGCAGGCTCAGC |

**S1 Table. Primers used in this study**
