# Supplementary material for: Development of a broad spectrum glycoconjugate vaccine to prevent wound and disseminated infections with Klebsiella pneumoniae and Pseudomonas aeruginosa
Source: PLoS One. 2018 Sep 6;13(9):e0203143. doi: 10.1371/journal.pone.0203143 (PMC6126813; doi:10.1371/journal.pone.0203143)
Supplement: S2 Table — (DOCX) [file pone.0203143.s005.docx]

| Flagellin type | Codon optimized sequence |
| --- | --- |
| A-type | ATGGCCCTGACGGTGAATACCAACATTGCGAGCCTGAATACGCAGCGCAACCTGAATAACAGCAGCGCGAGCCTGAATACGAGCCTGCAGCGTCTGAGCACGGGCAGCCGTATCAACAGCGCCAAAGATGATGCGGCCGGCCTGCAGATTGCGAATCGTCTGACGAGCCAGGTTAATGGCCTGAACGTGGCCACCAAAAATGCGAACGATGGCATCAGCCTGGCCCAGACCGCCGAAGGTGCACTGCAGCAGAGCACCAACATTCTGCAGCGCATGCGTGATCTGAGCCTGCAGAGCGCCAATGGCAGCAACAGCGATAGCGAACGCACCGCGCTGAACGGCGAAGTTAAACAGCTGCAGAAAGAACTGGATCGCATCAGCAATACCACGACCTTTGGTGGCCGTAAACTGCTGGATGGCAGCTTTGGCGTTGCGAGCTTTCAAGTGGGCAGCGCGGCCAACGAAATTATCAGCGTGGGCATTGATGAAATGAGCGCCGAAAGCCTGAATGGCACGTATTTTAAAGCCGATGGCGGCGGCGCAGTTACCGCCGCAACGGCCAGCGGCACGGTGGATATTGCCATTGGTATTACCGGCGGCAGCGCGGTGAATGTTAAAGTGGATATGAAGGGTAATGAAACGGCCGAACAGGCCGCGGCCAAAATTGCAGCGGCCGTTAATGATGCCAACGTGGGCATCGGCGCGTTTAGCGATGGCGATACGATTAGCTATGTTAGCAAAGCGGGCAAAGATGGCAGCGGTGCAATCACCAGCGCCGTGAGCGGCGTGGTTATTGCCGATACGGGCAGCACCGGCGTTGGTACCGCCGCCGGCGTGGCCCCGAGCGCCACCGCCTTTGCGAAAACGAACGATACCGTGGCCAAAATCGATATTAGCACCGCCAAAGGTGCACAGAGCGCCGTTCTGGTGATTGATGAAGCCATCAAACAGATTGATGCGCAGCGTGCCGATCTGGGTGCCGTTCAGAATCGTTTCGATAACACCATCAACAACCTGAAAAATATTGGTGAAAATGTGAGCGCCGCACGTGGTCGTATTGAAGATACCGATTTTGCGGCCGAAACGGCCAATCTGACCAAAAACCAGGTTCTGCAGCAGGCGGGTACGGCCATTCTGGCACAGGCGAATCAGCTGCCGCAGAGCGTGCTGAGCCTGCTGCGTTAA |
| B-type | ATGGCGCTGACGGTGAATACCAACATCGCCAGCCTGAACACGCAGCGCAATCTGAACGCCAGCAGCAATGATCTGAACACCAGCCTGCAGCGCCTGACCACGGGCTATCGTATTAACAGCGCGAAAGATGATGCGGCCGGCCTGCAGATTAGCAATCGCCTGAGCAACCAGATCAGCGGCCTGAATGTTGCGACCCGTAATGCCAACGATGGCATTAGCCTGGCCCAGACCGCCGAAGGTGCACTGCAGCAGAGCACCAATATTCTGCAGCGCATCCGTGATCTGGCGCTGCAGAGCGCCAATGGCAGCAACAGCGATGCGGATCGTGCGGCCCTGCAGAAAGAAGTGGCCGCACAGCAGGCGGAACTGACCCGTATTAGCGATACCACGACCTTTGGTGGCCGTAAACTGCTGGATGGCAGCTTTGGCACGACCAGCTTTCAGGTTGGCAGCAATGCCTATGAAACCATTGATATCAGCCTGCAGAATGCCAGCGCGAGCGCAATTGGCAGCTATCAAGTGGGCAGCAATGGTGCCGGTACCGTTGCGAGCGTGGCAGGCACCGCGACGGCCAGCGGCATTGCCAGCGGTACGGTTAATCTGGTGGGCGGCGGCCAGGTTAAAAACATTGCCATTGCCGCGGGTGATAGCGCCAAAGCAATTGCGGAAAAAATGGATGGTGCCATTCCGAATCTGAGCGCACGTGCCCGTACCGTGTTTACGGCAGATGTTAGCGGCGTGACCGGCGGCAGCCTGAATTTTGATGTGACGGTTGGCAGCAACACGGTTAGCCTGGCGGGCGTGACCAGCACCCAGGATCTGGCCGATCAGCTGAATAGCAACAGCAGCAAACTGGGCATTACGGCCAGCATCAATGATAAAGGCGTTCTGACCATCACGAGCGCCACCGGCGAAAACGTGAAATTTGGTGCACAGACGGGTACCGCAACGGCCGGTCAGGTGGCCGTTAAAGTGCAGGGCAGCGATGGCAAATTTGAAGCGGCCGCGAAAAATGTTGTTGCGGCCGGTACCGCGGCCACCACCACGATTGTTACCGGCTATGTGCAGCTGAACAGCCCGACGGCCTATAGCGTTAGCGGTACCGGTACCCAGGCGAGCCAGGTGTTTGGCAATGCCAGCGCCGCGCAGAAAAGCAGCGTTGCGAGCGTGGATATTAGCACGGCCGATGGTGCACAGAATGCAATTGCCGTGGTTGATAATGCGCTGGCCGCCATTGATGCCCAGCGCGCGGATCTGGGTGCGGTGCAGAACCGTTTCAAAAACACGATCGATAACCTGACCAACATCAGCGAAAATGCGACGAACGCCCGCAGCCGTATCAAAGATACGGATTTTGCCGCGGAAACCGCCGCGCTGAGCAAAAATCAGGTTCTGCAGCAGGCCGGTACGGCAATTCTGGCACAGGCCAACCAGCTGCCGCAGGCCGTGCTGAGCCTGCTGCGCTAA |

**S2 Table. Sequences of the codon optimized a-type and b-type flagellin cloned in pTrcHis - TOPO by TA cloning and produced as recombinant proteins in *E. coli* BL21**
